# Perceptions, symptoms, and practices of electronic cigarette users: Descriptive analysis and validation of Arabic short form vaping consequences questionnaire

**DOI:** 10.1371/journal.pone.0245443

**Published:** 2021-01-22

**Authors:** Reema Karasneh, Sayer Al-Azzam, Mohammad Nusair, Sahar Hawamdeh

**Affiliations:** 1 Department of Basic Medical Sciences, Faculty of Medicine, Yarmouk University, Irbid, Jordan; 2 Clinical Pharmacy Department, Faculty of Pharmacy, Jordan University of Science and Technology, Irbid, Jordan; 3 Department of Pharmacy Practice, Faculty of Pharmacy, Yarmouk University, Irbid, Jordan; University of Calfornia San Francisco, UNITED STATES

## Abstract

**Background:**

E-cigarette (EC) use is increasing worldwide. Understanding the practices and perceptions of e-cigarette users and profiling the symptoms they experience is essential for regulating the use of such products. This study aims to investigate the practices and perceptions of e-cigarette users in Jordan and examine the symptoms (e.g. respiratory) they associate with e-cigarette use.

**Methods:**

A cross-sectional online survey was conducted to assess EC use and tobacco smoking behaviors and the corresponding health symptoms among EC users in Jordan. EC use expectancies were also assessed using the Short Form Vaping Consequences Questionnaire, which was first translated into Arabic and tested for validity and reliability.

**Results:**

Out of the 400 EC users surveyed, 95.5% were male, 76.2% used nicotine-containing juice, and 56.8% were concurrent tobacco smokers. Further, the participants had a mean age of 28.9 years (±10.2). Among dual EC/cigarette users, 88.6% reported that they tried to quit cigarette smoking, with e-cigarette use being the most commonly tried method of smoking cessation. The smoking-related symptoms reported by regular cigarette smokers mainly included sputum production (77.5%). The participants reported that using e-cigarettes instead of tobacco cigarettes had led to improvements in their sputum production (60.8%), breathing (59%), and general wellbeing (52%). Pleasant taste, enjoyable taste sensation, and flavor were significantly stronger (*P*-value < 0.05) among e-cigarette users compared to dual users. Dual EC/cigarette users reported stronger perceptions in the negative consequences scale, particularly with regards to the hazardous effects of smoking on health (*P*-value < 0.05).

**Conclusion:**

Dual daily use of e-cigarettes and regular cigarettes is a common practice among EC users. We recommend that further research is conducted on dual EC/cigarette use and the potential health risks this may have (e.g. higher nicotine intake as compared to the single use of either products).

## Introduction

In recent years, there has been a marked increase in e-cigarette (EC) use globally. The e-cigarette market has witnessed notable growth over the past few years, reaching a value of around $2.5 billion, a number which is yet expected to rise [1,2]. In light of this rapid growth, many controversies have emerged concerning the safety and health issues associated with e-cigarette use.

An e-cigarette is an electronic nicotine delivery system where, unlike conventional cigarettes, no tobacco combustion takes place. Rather, a special solution that may or may not contain nicotine, in addition to flavorings and other ingredients such as propylene glycol and glycerine, is heated to form an aerosol or 'vapor' that is inhaled by the user. The product is designed to mimic the psychological experience of conventional cigarette smoking and is claimed to cause less harm and exposure to toxic constituents [3]. However, there is no conclusive evidence to strongly support these claims [4]. Although many of the harmful chemicals found in tobacco smoke are not present in significant amounts in EC solutions, the Food and Drug Association (FDA) argues that detectable levels of certain carcinogens have been measured [5]. The toxic constituents in EC solutions reported by the FDA include nitrosamines and diethylene glycol, among many others. Moreover, with the emergence of EC-associated acute lipoid pneumonia case reports, the possible deleterious effects of EC use have recently received public attention [6,7]. Furthermore, EC use has been suggested to impact cardiovascular health, as users of nicotine-containing e-cigarettes have been observed to experience an increase in systolic blood pressure and heart rate for about 45 minutes following EC use [8].

With tobacco smoking being a major risk factor for a myriad of diseases and a leading cause of preventable death worldwide, the use of e-cigarettes as a tool for reducing the harm associated with tobacco smoking is often perceived to be justifiable [9]. This calls for a clear understanding of the practices, benefits, and risks associated with EC use. Further, the issue of EC safety is not confined to the presence or lack of toxic constituents, as it can be argued that tobacco morbidity and mortality do not justify the replacement of one method of nicotine delivery with another. Rather, efforts should be focused on nicotine abstinence altogether [10].

Several studies have shown the majority of EC users to be former or current tobacco smokers [11–13]. In these studies, the reported motives behind engaging in EC use have varied, including the desire to reduce the harmful effects of cigarette smoking, decrease cigarette consumption, or quit smoking. Other reasons have included the reduced environmental harm and second-hand smoking effects caused by EC use as compared to conventional cigarette smoking.

There has also been a considerable spread of EC use among never-smokers, a trend particularly prominent among youth [14]. In a study by Sutfin et al., 216 out of 4444 surveyed college students reported being current or former EC users, and whilst EC use was more common among former cigarette smokers, 12% of EC users were never-smokers. Users may also find e-cigarettes to be convenient in allowing them to smoke in smoke-free areas [15]. Other motivations for EC use include the belief that they are healthier and cheaper than cigarettes [16].

It is important to understand EC users’ perceptions and patterns of EC use in order to address the health impacts associated with EC use and guide EC sale regulations. Studies in the literature have mainly been based on online surveys targeted at EC website visitors, and little is known about the population of EC users in the Middle East. A very recent study in Lebanon investigated EC-related knowledge and attitudes among a cross-sectional sample [17]. However, despite the high prevalence of EC use in the region, no studies have assessed the practices of EC users in the Middle East. Recent studies have reported that in Jordan, where the current study was conducted, the prevalence of EC use ranges between 11.7%-18% [18,19]. Further, in a study conducted among college students in Saudi Arabia, an EC use prevalence of 27.7% was reported [20]. Therefore, the present study aimed to investigate the demographic characteristics, usage patterns, and EC-related health perceptions among EC users in Jordan. The findings of this study can be used to guide future research related to EC use in the Middle East, as well as ultimately guiding EC regulation in the region.

## Methods

### Study design

A cross‐sectional survey was conducted from February to May 2019 among adult EC users in Jordan. An online self‐administered questionnaire was adapted from the Short Form Smoking Consequences Questionnaire (S-SCQ) [21]. The S-SCQ was reworded to S-VCQ to reflect EC expectancies [22]. Ethical approval for conducting this study was obtained from the Institutional review boards of King Abdullah University Hospital (reference number 35/120/2019). All performed procedures were in accordance with the ethical standards of the institutional research committee and with the 1964 Helsinki declaration and its later amendments or comparable ethical standards. Electronic informed consent for participation in this study was obtained from the participants, whilst no personally identifiable information was obtained.

### Survey instrument

The online survey consisted of four sections. The first section comprised six items related to the demographic characteristics of the respondents, while the second section comprised 11 questions aimed at identifying the respondents’ EC and tobacco smoking behaviors. The third section was aimed at identifying the symptoms that the respondents experienced as a result of smoking regular cigarettes and whether these symptoms had improved after they had started using e-cigarettes. Finally, the fourth section included the S-VCQ, which comprises 21 items aimed at assessing EC use outcome expectancies and which has been evidenced to be valid and reliable for use among adults and adolescents [21,22]. In order for it to be usable among adults in Jordan, the S-VCQ was translated into Arabic following ISPOR’s Principles of Good Practice for Translation and Cultural Adaptation [23]. In the cognitive debriefing phase of the translation, the Arabic S-VCQ was tested on a sample of 18 adults, an essential step for ensuring the understandability, interpretation, and cultural relevance of the translated survey. During this stage, the participants reported that some questions were similar in meaning and thus needed to be eliminated to avoid confusion. Therefore, in the cognitive debriefing review, the authors removed four items, resulting in a total of 17 questions in the final version of the Arabic S-VCQ. A review of the final version of the S-VCQ was then conducted by a professor specialized in the Arabic language in order to check for any typographical or grammatical mistakes (S1 File).

### Data collection

To this date, e-cigarettes are not legally allowed to be sold in Jordan, and the majority of EC and EC accessory sales are thus conducted through social media networks. Therefore, participants were invited to take part in this study through social media EC groups and pages. The survey was administered using Survey Monkey, an online survey collection tool, and had a required completion time of approximately 10 minutes. In order to ensure that there were no missing data, all of the survey items were “required”. At the beginning of the survey, all respondents were informed about the study purposes and assured that their participation was completely voluntary and anonymous, and that all data would be treated as confidential. The anonymous survey model was implemented to ensure confidentiality and to remedy the potential effect of social desirability bias.

### Data analysis

The survey responses were analyzed using the Statistical Package for the Social Sciences (SPSS) version 25. Confirmatory factor analysis was performed to assess the construct validity of the adapted Arabic S-VCQ. Cronbach's alpha coefficient was used to assess the reliability of the adapted survey, with an alpha value of ≥ 0.7 considered satisfactory [24]. Furthermore, descriptive statistical analyses were used to compare between the results of the tobacco smoker and nonsmoker groups. Statistical significance was tested using the Chi-square test for the categorical variables and unpaired t-test for the continuous variables.

## Results

A total of 400 EC users participated in this study, with a median age of 28 years (range 18–47 years) among the participants. The majority of the participants (95.5%) were male (Table 1), and all participants were former tobacco cigarette smokers. Most of the participants did not suffer from any chronic medical conditions (92.2%, n = 369).

**Table 1:** Participants’ characteristics.

Demographic Variables (n = 400)		N (%)
Gender		
	Male	382 (95.5)
	Female	18 (4.5)
Education		
	Secondary	61 (15.8)
	Post-Secondary	339 (84.2)
Employment		
	Employed	288 (72)
	Unemployed	112 (28)
Chronic Conditions		
	Yes	31 (7.8)
	No	369 (92.2)
E-Cigarette Smoking Style		
	Dual smoker (tobacco and e-cigarette)	227 (56.8)
	E-cigarette only	173 (43.2)
Age		
	Median (range)	28 (18–47)

### Regular cigarette smoking

Of the participating EC users, 56.8% (n = 227) were dual cigarette smokers (i.e., tobacco and EC). Most dual smokers reported smoking regular cigarettes on a daily basis, with the majority smoking one or more packs per day (Table 2). Further, most of the participants (87.3%) had previously attempted to quit cigarette smoking, with 62% having tried to quit more than once. The reported methods adopted for quitting regular cigarettes included EC use (66.8%), nicotine gums (2.6%), nicotine patches (2.6%), prescription medications (e.g., bupropion) (0.9%), and going cold turkey (27.1%). The overwhelming majority of the participants reported that they planned to quit smoking at the time of the survey (89.4%, n = 203).

**Table 2:** Cigarette smoking habits among dual users.

Cigarette Smoking Habits (n = 227)		
Cigarette Smoking Frequency		
	Daily	190 (83.0)
	Occasionally/Socially	39 (17.0)
Number of Cigarettes/Day		
	<5 cigarettes	34 (14.8)
	5–9 cigarettes	13 (5.7)
	10–14 cigarettes	21 (9.1)
	15–19 cigarettes	19 (8.3)
	One pack	81 (35.4)
	More than one pack	61 (26.7)
Tried to Quit		
	Never	29 (12.7)
	Once	58 (25.3)
	More than once	142 (62)
Smoking Cessation Method		
	Nicotine gum	6 (2.6)
	Nicotine patches	6 (2.6)
	Prescription medications	2 (0.9)
	E-cigarette	153 (66.8)
	Cold turkey	62 (27.1)
Planning to Quit		
	Yes	203 (89.4)
	No	24 (10.6)

### E-cigarette use patterns

The participants’ EC use patterns were assessed (Table 3). Most of the participants (77.8%) reported using e-cigarettes on a regular basis, whilst 22.2% reported using e-cigarettes occasionally or socially. Further, 67% reported having used e-cigarettes for six months or less. The results showed that on a typical day, EC-only users had significantly more puffs than did dual cigarette users (P = 0.049). Moreover, EC-only users used nicotine free juices significantly more often than did dual cigarette users (P<0.001; Table 3).

**Table 3:** E-cigarette use patterns among the study participants (n = 400).

Practice			N (%)	Dual cigarette users N (%)	E-cigarette only users N(%)	P-value
**How long have you been using e-cigarettes**						
	3 months or less		198 (49.5)	142 (62.6)	56 (32.4)	<0.001
	6 months		70 (17.5)	35 (15.4)	35 (20.2)	
	12 months		33 (8.3)	13 (5.7)	20 (11.6)	
	More than 12 months		99 (24.8)	37 (16.3)	62 (35.8)	
**How often do you use e-cigarettes**						
	On a regular basis		311 (77.8)	177 (78)	134 (77.5)	0.904
	Occasionally/Socially		89 (22.2)	50 (22)	39 (22.5)	
**Number of puffs/days**						
	< 50		93 (23.3)	60 (26.4)	33 (19.1)	0.049
	50–99		140 (35.0)	78 (34.4)	62 (35.8)	
	100–150		77 (19.2)	48 (21.2)	29 (16.8)	
	> 150		90 (22.5)	41 (18.1)	49 (28.3)	
**Type of juice**						
	With Nicotine		305 (76.2)	193 (85)	112 (64.7)	<0.001
	Nicotine Free		35 (8.8)	10 (4.4)	25 (14.5)	
	Both		60 (15.0)	24 (10.6)	36 (20.8)	
**First e-cigarette puff**						
	As soon as I wake up		86 (21.5)	51 (22.5)	35 (20.2)	0.104
	30–60 minutes after I wake up		139 (34.8)	87 (38.3)	52 (30.1)	
	> 60 minutes after I wake up		175 (43.8)	89 (39.2)	86 (49.7)	
**Type of e-cigarette**						
	Mini EC		263 (59)	162 (71.4)	101 (58.4)	0.008
	Mid-size EC Mods		39 (8.7)	18 (7.9)	21 (12.1%)	0.109
	Customizable EC Mods		144 (32.3)	60 (26.4)	84 (48.6%)	<0.001

* Pearson’s Chi-squared test and Fisher’s exact test were used.

The most common type of juice used among the participants was nicotine-containing juice (76.2%). However, only 15% of EC users reported using both nicotine-containing and nicotine-free juices. A considerable percentage of the participating EC users (21.5%) reported taking their first EC puff as soon as they woke up, although 43.8% reported that they waited at least an hour after waking up. The most commonly used type of EC among the participating EC users was the mini EC (59%), with customizable EC mods being significantly (P<0.001) more preferable to EC-only users (48.6%, n = 84).

### Symptoms

The smoking-related symptoms experienced by the participants were assessed (Table 4). Sputum production was the symptom that was most commonly reported to be experienced by the participants when they smoked regular cigarettes. Interestingly, 12% of the participants who smoked tobacco cigarettes reported experiencing no cigarette-associated symptoms, such as coughing. Further, participants who had transitioned from smoking tobacco cigarettes to using e-cigarettes reported that they had experienced improvements in sputum production (60.8%), breathing (59%), and general wellbeing (52%).

**Table 4:** Self-reported symptoms that were aggravated by regular cigarette smoking and self-reported symptoms that had improved after the participants had started EC use (n = 400).

Symptoms that the participants suffered from when they smoked regular cigarettes	N (%)	Dual cigarette users N (%)	E-cigarette only users N (%)	P-value
Chest wheezing	155 (38.8)	101 (44.5)	54 (31.2)	0.007
Shortness of breath	211 (52.8)	130 (57.3)	81 (46.8)	0.043
Sputum production	268 (67.0)	176 (77.5)	92 (53.3)	<0.001
Cough	218 (54.5)	138 (60.8)	80 (46.2)	0.005
Nothing	48 (12.0)	26 (11.5)	22 (12.7)	0.757
Other symptoms (i.e., chest discomfort, headaches, blurred vision, and stomach upset)	8 (2.0)	4 (1.8)	4 (2.3)	0.731
**Symptoms that had improved after the participants had started EC use**	**N (%)**			
Cough	192 (48.0)	112 (49.3)	80 (46.2)	0.546
General well-being	210 (52.5)	118 (52.0)	92 (53.2)	0.840
Breathing	224 (56.0)	134 (59.0)	90 (52.0)	0.186
Sputum production	228 (57.0)	138 (60.8)	90 (52.0)	0.046
Other symptoms (i.e., increased appetite was reported)	1 (0.3)	1 (0.4)	0 (0.0)	1.000

* Pearson’s Chi-squared test and Fisher’s exact test were used.

### Validity and reliability

In order to ensure that the Arabic S-VCQ would yield constructs similar to the original S-VCQ, the construct validity of the Arabic S-VCQ was assessed using confirmatory factor analysis [25]. Four factors emerged from the factor analysis: positive reinforcement, negative reinforcement, appetite/weight control, and negative consequences (Table 5). The cumulative variance of the four-factor solution was 68.34%. The Kaiser-Meyer Olkin (KMO) test and Bartlett’s test of sphericity were used to verify the adequacy of the sampling and examine the appropriateness of the factor analysis. The Arabic S-VCQ obtained a good KMO measure (0.842), with a significant Bartlett’s test of sphericity result (P <0.001) [26]. The Cronbach's alpha coefficients for the four factors ranged from 0.718 to 0.909, indicating that the Arabic S-VCQ had acceptable to excellent internal reliability [27].

**Table 5:** Dual cigarette users and e-cigarette only users’ perceptions towards e-cigarette use and factor analysis of the Arabic S-VCQ.

Item	Total (mean ± SD)	Dual cigarette users (mean ± SD)	E-cigarette only users (mean ± SD)	P-value *	Factor			
					1	2	3	4
**Positive Reinforcement Scale (Cronbach α = 0.831)**								
E-cigarettes taste good	8.3±1.93	8.1±2.1	8.7 ± 1.63	0.003		0.625		
I enjoy the taste sensation I experience while using e-cigarettes	8.4 ±1.69	8.1±1.86	8.8 ± 1.36	<0.001		0.832		
When I use e-cigarettes, the taste is pleasant	8.1 ± 1.98	8.1 ± 1.91	8.2 ± 2.07	0.627		0.723		
I enjoy the flavor of e-cigarettes	8.5 ± 1.66	8.3 ± 1.7	8.8 ± 1.56	0.001		0.890		
I enjoy feeling the e-cigarette on my tongue and lips	8.0 ± 2.14	7.9 ± 2.14	8.0 ± 2.15	0.565		0.760		
**Negative Reinforcement Scale (Cronbach α = 0.909)**								
E-cigarettes help me deal with my anxiety	5.5±2.81	5.5±2.78	5.6 ± 2.86	0.817	0.694			
E-cigarettes help me deal with my depression	4.5 ± 2.98	4.4±2.93	4.8 ± 3.04	0.191	0.670			0.328
E-cigarettes help me reduce or handle tension	4.5 ± 2.74	4.5 ± 2.72	4.5 ± 2.8	0.884	0.754			
When I am upset with someone, the e-cigarette helps me cope	4.9 ± 2.74	4.8 ± 2.72	5.1 ± 2.77	0.321	0.825			
E-cigarette use calms me down when I feel nervous	5.5 ±2.66	5.4 ± 2.6	5.6 ± 2.75	0.558	0.900			
When I am angry, an e-cigarette can calm me down	5.4 ± 2.61	5.3 ± 2.51	5.5 ± 2.73	0.308	0.912			
**Appetite/Weight Control Scale (Cronbach α = 0.718)**								
E-cigarette use helps me control my appetite	4.8± 3.07	4.9±3.07	4.6 ± 3.08	0.478				0.760
E-cigarette use keeps me from eating more than I should	4.0 ± 2.67	4.1 ± 2.62	3.8 ± 2.72	0.249	0.386			0.769
E-cigarette use helps me keep my weight down	3.6 ± 2.55	3.6 ± 2.42	3.5 ± 2.72	0.655	0.401			0.691
**Negative Consequences Scale (Cronbach α = 0.814)**								
E-cigarette use puts me at risk of heart disease and lung cancer	4.0 ± 2.81	4.1 ± 2.85	3.8 ± 2.76	0.371			0.857	
E-cigarette use is hazardous to my health	4.8 ± 2.88	5.1 ± 2.9	4.5 ± 2.76	0.025			0.841	
E-cigarette use is taking years off my life	3.7 ± 2.77	3.8 ± 2.8	3.5 ± 2.73	0.235			0.830	

* The unpaired t-test was used to compare the mean scores between the ‘dual cigarette users’ group and the ‘e-cigarette only users’ group

### Perceptions

The validated Arabic S-VCQ was used to assess dual cigarette users and EC-only users’ perceptions towards EC use (Table 5). In the positive reinforcement scale, perceptions regarding good taste, enjoyable taste sensation, and EC flavor were significantly stronger (p-value < 0.05) among EC-only users. On the other hand, dual cigarette users reported stronger perceptions in the negative consequences scale than did EC-only users, particularly regarding the hazardous effects of EC use on health (P = 0.025). Meanwhile, no differences were identified between dual cigarette users and EC-only users in the negative reinforcement and appetite/weight control scales.

## Discussion

The majority of the EC users in the current study were also tobacco smokers who smoked both regular cigarettes and e-cigarettes on a daily basis. This finding is similar to the findings reported in other studies conducted among EC users, although the percentage of dual users was higher in our study [11–13, 29, 30]. Whilst 56.8% of the EC users in our study were also cigarette smokers, two similar studies reported percentages of 11.5% and 18% [28,29]. Considering that many of the participants reported having previously used e-cigarettes to aid tobacco cessation, dual use may have resulted from failed cessation attempts. Other researchers have proposed that reduction in costs and the ability to use e-cigarettes in smoke-free areas may be motives for the dual use of regular cigarettes and e-cigarettes [30]. In addition, dual users may desire to achieve a balance between the 'real' experience of tobacco smoking and the reduced harm of EC use [30]. However, we believe that this is not the case among our sample, as dual use was associated with a stronger perception that EC use is hazardous to health. Further, we found that dual use had detrimental effects on the participants, whereby dual users reported experiencing chest wheezing, sputum production, and cough more often than did EC-only users. Similarly, a study among 7505 males found dual users to be more nicotine-dependent as compared to cigarette-only smokers [31].

In terms of EC juice preference, most of our study participants reported using nicotine-containing EC juices. However, EC-only users used nicotine-free juices significantly more often than did dual users, which supports the idea that dual use is not always a temporary phase that leads to the ultimate goal of quitting tobacco cigarettes. Rather, dual use may often end up being a long-term practice. The majority of EC users in similar studies have also been reported to prefer nicotine-containing solutions [11,12,32], with tobacco smokers and heavy EC users preferring medium to high nicotine concentrations [33,34]. This preference of nicotinic juices raises concerns regarding the safety of EC use. According to the American Heart Association, the harmful effects of nicotine addiction may include "hemodynamic effects, endothelial dysfunction, thrombogenesis, systemic inflammation, and other metabolic effects" [35].

Flavor was also found to be an important factor impacting the participants’ choice of EC juice, as certain flavors may encourage usage or be associated with increased or reduced harm perception [32]. A study by Kroemer et al. also showed sweet-flavored e-cigarette juices to be associated with increased brain cue-reactivity and the potentiation of the reinforcing effects of nicotine, thus increasing appeal [36]. Our findings indicate that good taste and flavor have strong positive reinforcement effects among EC users, particularly EC-only users. Flavor plays a role in creating the sensory effect of EC use, which may increase appeal for EC use among never-smokers [37]. In fact, the fact that EC juices come in different flavors was reported as being a main reason for EC use by 81% of youth in a study conducted between 2013 and 2014 [38]. Therefore, it is important to consider flavor when regulating the sales and availability of EC products and accessories in the market.

The participants in our study reported improvement in health outcomes as a result of switching from regular cigarettes to e-cigarettes, particularly with regards to sputum production, breathing, coughing, and general wellbeing. Similarly, respondents to a previous internet survey listed better health outcomes as an advantage of switching from tobacco smoking to EC use [39] This improvement in general health may have an impact on EC users' health perceptions. Our findings are consistent with the findings of several studies that reported improvement in health perceptions among smokers who had switched from conventional cigarettes to e-cigarettes or who were using e-cigarettes for smoking reduction. In a study by Van et al., which targeted online EC shop customers, 84% of EC users reported that they had experienced improvements in their general health after switching to EC products [40]. Similarly, another survey among EC users revealed a significant association between switching to EC use and better-perceived health, especially when cigarette use is reduced by more than 2 packets/month [28]. Additionally, EC use was believed to aid weight control among the respondents in our study, and no significant differences were found between dual and single users in the appetite/weight control scale. This is similar to the findings of several studies, including a study in the United Kingdom which reported that e-cigarettes are often used to replace a meal or a snack [41–44].

The present study is one of few studies conducted among EC users in the Middle East, where EC use is on the rise. The study also captures the overlap between tobacco cigarette smoking and EC use and provides useful data on EC use patterns, especially dual use. However, there are a few limitations to the current study. For example, online recruitment may have led to selection bias in favor of younger EC users who regularly use social media platforms. However, considering the fact that most EC sales in Jordan are conducted through social media platforms, this may not have affected our findings. Finally, since this study used a self-reported questionnaire, certain parameters could not be verified, such as the presence of chronic conditions.

## Conclusion

With the global rise in EC use and the ambiguity surrounding the safety profiles of such products, it is of paramount importance to explore EC use patterns, perceptions, and attitudes among users. Our findings have revealed that there is a high prevalence of the dual use of tobacco cigarettes and e-cigarettes in Jordan. Further, concerning practices were identified among the participants, such as the daily use of both tobacco cigarettes and e-cigarettes by most users and the preference for nicotine-containing e-juices. Future studies with extended follow-up periods are required to investigate the transition from regular cigarettes to e-cigarettes or vice versa.

## Supporting information

S1 File Arabic short form vaping consequences questionnaire. (DOCX)

## References

[1] Giovenco DP, Hammond D, Corey CG, Ambrose BK, Delnevo CD. E-Cigarette market trends in traditional U.S. retail channels, 2012–2013. Nicotine Tob Res. 2015;17: 1279–1283. 10.1093/ntr/ntu282 25542918 PMC4683368

[2] King BA, Alam S, Promoff G, Arrazola R, Dube SR. Awareness and ever-use of electronic cigarettes among U.S. adults, 2010–2011. Nicotine Tob Res. 2013;15: 1623–1627. 10.1093/ntr/ntt013 23449421 PMC4570561

[3] Yule JA, Tinson JS. Youth and the sociability of “Vaping.” J Consum Behav. 2017;16: 3–14. 10.1002/cb.1597

[4] Douglass B, Solecki S, Fay-Hillier T. The Harmful Consequences of Vaping: A Public Health Threat. J Addict Nurs. 2020;31: 79–84. 10.1097/JAN.0000000000000332 32487933

[5] Drummond MB, Upson D. Electronic cigarettes: Potential harms and benefits. Ann Am Thorac Soc. 2014;11: 236–242. 10.1513/AnnalsATS.201311-391FR 24575993 PMC5469426

[6] Davidson K, Brancato A, Heetderks P, Mansour W, Matheis E, Nario M, et al Outbreak of Electronic-Cigarette–Associated Acute Lipoid Pneumonia—North Carolina, July–August 2019. MMWR Morb Mortal Wkly Rep. 2019;68: 784–786. 10.15585/mmwr.mm6836e1 31513559 PMC6755817

[7] Schier JG, Meiman JG, Layden J, Mikosz CA, VanFrank B, King BA, et al Severe Pulmonary Disease Associated with Electronic-Cigarette–Product Use—Interim Guidance. MMWR Morb Mortal Wkly Rep. 2019;68: 787–790. 10.15585/mmwr.mm6836e2 31513561 PMC6755818

[8] Franzen KF, Willig J, Cayo Talavera S, Meusel M, Sayk F, Reppel M, et al E-cigarettes and cigarettes worsen peripheral and central hemodynamics as well as arterial stiffness: A randomized, double-blinded pilot study. Vasc Med (United Kingdom). 2018;23: 419–425. 10.1177/1358863X18779694 29985113

[9] E-Cigarettes are a US$2 Billion Global Industry.

[10] Palazzolo DL. Electronic cigarettes and vaping: A new challenge in clinical medicine and public health. A literature review. Front Public Heal. 2013;1: 56 10.3389/fpubh.2013.00056 24350225 PMC3859972

[11] Etter JF, Bullen C. Electronic cigarette: Users profile, utilization, satisfaction and perceived efficacy. Addiction. 2011;106: 2017–2028. 10.1111/j.1360-0443.2011.03505.x 21592253

[12] Regan AK, Promoff G, Dube SR, Arrazola R. Electronic nicotine delivery systems: Adult use and awareness of the “e-cigarette” in the USA. Tob Control. 2013;22: 19–23. 10.1136/tobaccocontrol-2011-050044 22034071

[13] Sutfin EL, McCoy TP, Morrell HER, Hoeppner BB, Wolfson M. Electronic cigarette use by college students. Drug Alcohol Depend. 2013;131: 214–21. 10.1016/j.drugalcdep.2013.05.001 23746429 PMC3760168

[14] Carroll Chapman SL, Wu LT. E-cigarette prevalence and correlates of use among adolescents versus adults: A review and comparison. J Psychiatr Res. 2014;54: 43–54. 10.1016/j.jpsychires.2014.03.005 24680203 PMC4055566

[15] Glantz SA, Bareham DW. E-Cigarettes: Use, Effects on Smoking, Risks, and Policy Implications. Annual Review of Public Health. 2018 10.1146/annurev-publhealth-040617-013757 29323609 PMC6251310

[16] Sussan TE, Shahzad FG, Tabassum E, Cohen JE, Wise RA, Blaha MJ, et al Electronic cigarette use behaviors and motivations among smokers and non-smokers. BMC Public Health. 2017 10.1186/s12889-017-4671-3 28882123 PMC5590123

[17] Aghar H, El-Khoury N, Reda M, Hamadeh W, Krayem H, Mansour M, et al Knowledge and attitudes towards E-cigarette use in Lebanon and their associated factors. BMC Public Health. 2020;20: 278 10.1186/s12889-020-8381-x 32111186 PMC7049178

[18] Al-Balas HI, Al-Balas M, Al-Balas H, Almehaiza S, Melhem HB, Al-Balas B. Electronic Cigarettes Prevalence and Awareness Among Jordanian Individuals. J Community Health. 2020 10.1007/s10900-020-00904-x 32776292

[19] Abdel-Qader DH, Al Meslamani AZ. Knowledge and Beliefs of Jordanian Community Toward E-cigarettes: A National Survey. J Community Health. 2020 10.1007/s10900-020-00896-8 32772206

[20] Qanash S, Alemam S, Mahdi E, Softah J, Touman AA, Alsulami A. Electronic cigarette among health science students in Saudi Arabia. Ann Thorac Med. 2019;14: 56–62. 10.4103/atm.ATM_76_18 30745936 PMC6341860

[21] Myers MG, MacPherson L, McCarthy DM, Brown SA. Constructing a short form of the Smoking Consequences Questionnaire with adolescents and young adults. Psychol Assess. 2003;15: 163–172. 10.1037/1040-3590.15.2.163 12847776 PMC1855290

[22] Morean ME, L’Insalata A. The Short Form Vaping Consequences Questionnaire: Psychometric Properties of a Measure of Vaping Expectancies for Use With Adult E-cigarette Users. Nicotine Tob Res. 2017;19: 215–221. 10.1093/ntr/ntw205 27613904

[23] Wild D, Grove A, Martin M, Eremenco S, McElroy S, Verjee-Lorenz A, et al Principles of Good Practice for the Translation and Cultural Adaptation Process for Patient-Reported Outcomes (PRO) Measures: Report of the ISPOR Task Force for Translation and Cultural Adaptation. Value Heal. 2005;8: 94–104. 10.1111/j.1524-4733.2005.04054.x 15804318

[24] Bland JM, Altman DG. Cronbach’s alpha. BMJ. 1997;314: 572 10.1136/bmj.314.7080.572 9055718 PMC2126061

[25] Williams B, Onsman A, Brown T. Exploratory factor analysis: A five-step guide for novices EDUCATION Exploratory factor analysis: A five-step guide for novices. Australas J Paramed. 2012.

[26] Spicer J. Making Sense of Multivariate Data Analysis: An Intuitive Approach. Making Sense of Multivariate Data Analysis: An Intuitive Approach. 2004.

[27] Tavakol M, Dennick R. Making sense of Cronbach’s alpha. International journal of medical education. 2011 10.5116/ijme.4dfb.8dfd 28029643 PMC4205511

[28] Hart JL, Walker KL, Sears CG, Lee AS, Ridner SL, Keith RJ. E-cigarette use and perceived health change: Better health through vaping? Tob Induc Dis. 2018;16: 48 10.18332/tid/95218 31516445 PMC6664314

[29] Yingst JM, Veldheer S, Hrabovsky S, Nichols TT, Wilson SJ, Foulds J. Factors associated with electronic cigarette users’ device preferences and transition from first generation to advanced generation devices. Nicotine Tob Res. 2015;17: 1242–1246. 10.1093/ntr/ntv052 25744966 PMC4592341

[30] Robertson L, Hoek J, Blank ML, Richards R, Ling P, Popova L. Dual use of electronic nicotine delivery systems (ENDS) and smoked tobacco: A qualitative analysis. Tob Control. 2019;28: 13–19. 10.1136/tobaccocontrol-2017-054070 29419488 PMC6317506

[31] Kim C-Y, Paek Y-J, Seo HG, Cheong YS, Lee CM, Park SM, et al Dual use of electronic and conventional cigarettes is associated with higher cardiovascular risk factors in Korean men. Sci Rep. 2020;10: 5612 10.1038/s41598-020-62545-3 32221375 PMC7101350

[32] Zare S, Nemati M, Zheng Y. A systematic review of consumer preference for e-cigarette attributes: Flavor, nicotine strength, and type. Cormet-Boyaka E, editor. PLoS One. 2018;13: e0194145 10.1371/journal.pone.0194145 29543907 PMC5854347

[33] Morean ME, Kong G, Cavallo DA, Camenga DR, Krishnan-Sarin S. Nicotine concentration of e-cigarettes used by adolescents. Drug Alcohol Depend. 2016;167: 224–227. 10.1016/j.drugalcdep.2016.06.031 27592270 PMC5158305

[34] Goldenson NI, Leventhal AM, Stone MD, McConnell RS, Barrington-Trimis JL. Associations of electronic cigarette nicotine concentration with subsequent cigarette smoking and vaping levels in adolescents. JAMA Pediatr. 2017;171: 1192–1199. 10.1001/jamapediatrics.2017.3209 29059261 PMC5779618

[35] Piano MR, Benowitz NL, Fitzgerald GA, Corbridge S, Heath J, Hahn E, et al Impact of smokeless tobacco products on cardiovascular disease: implications for policy, prevention, and treatment: a policy statement from the American Heart Association. Circulation. 2010;122: 1520–44. 10.1161/CIR.0b013e3181f432c3 20837898

[36] Kroemer NB, Veldhuizen MG, Delvy R, Patel BP, O’Malley SS, Small DM. Sweet taste potentiates the reinforcing effects of e-cigarettes. Eur Neuropsychopharmacol. 2018;28: 1089–1102. 10.1016/j.euroneuro.2018.07.102 30093174

[37] Schneller LM, Bansal-Travers M, Goniewicz ML, McIntosh S, Ossip D, O’Connor RJ. Use of Flavored E-Cigarettes and the Type of E-Cigarette Devices Used among Adults and Youth in the US-Results from Wave 3 of the Population Assessment of Tobacco and Health Study (2015–2016). Int J Environ Res Public Health. 2019;16 10.3390/ijerph16162991 31434229 PMC6720922

[38] Villanti AC, Johnson AL, Ambrose BK, Cummings KM, Stanton CA, Rose SW, et al Flavored Tobacco Product Use in Youth and Adults: Findings From the First Wave of the PATH Study (2013–2014). Am J Prev Med. 2017;53: 139–151. 10.1016/j.amepre.2017.01.026 28318902 PMC5522636

[39] Etter JF. Electronic cigarettes: A survey of users. BMC Public Health. 2010;10: 231 10.1186/1471-2458-10-231 20441579 PMC2877672

[40] Van Gucht D, Adriaens K, Baeyens F. Online vape shop customers who use e-cigarettes report abstinence from smoking and improved quality of life, but a substantial minority still have vaping-related health concerns. Int J Environ Res Public Health. 2017;14: 798 10.3390/ijerph14070798 28714914 PMC5551236

[41] Glover M, Breier BH, Bauld L. Could Vaping be a New Weapon in the Battle of the Bulge? Nicotine Tob Res. 2016;19: ntw278 10.1093/ntr/ntw278 27798086

[42] Morean ME, Wedel A V. Vaping to lose weight: Predictors of adult e-cigarette use for weight loss or control. Addict Behav. 2017;66: 55–59. 10.1016/j.addbeh.2016.10.022 27875790

[43] Bennett BL, Pokhrel P. Weight Concerns and Use of Cigarettes and E-Cigarettes among Young Adults. Int J Environ Res Public Health. 2018;15 10.3390/ijerph15061084 29843377 PMC6024963

[44] Jackson SE, Brown J, Aveyard P, Dobbie F, Uny I, West R, et al Vaping for weight control: A cross-sectional population study in England. Addict Behav. 2019;95: 211–219. 10.1016/j.addbeh.2019.04.007 30981033 PMC6555398

