# Supplementary material for: Perceptions, symptoms, and practices of electronic cigarette users: Descriptive analysis and validation of Arabic short form vaping consequences questionnaire
Source: PLoS One. 2021 Jan 22;16(1):e0245443. doi: 10.1371/journal.pone.0245443 (PMC7822314; doi:10.1371/journal.pone.0245443)
Supplement: S1 File — (DOCX) [file pone.0245443.s001.docx]

**Arabic Short Form Vaping Consequences Questionnaire**

**استبيان تبعات التدخين الالكتروني – النموذج القصير**

**الرجاء وضع دائرة على الخيار الأقرب للصحة باعتقادك وحسب المقياس أدناه.**

| **0** | **1** | **2** | **3** | **4** | **5** | **6** | **7** | **8** | **9** |
| --- | --- | --- | --- | --- | --- | --- | --- | --- | --- |
| **كلياً** | **إلى حد كبير** | **كثيراً** | **إلى حد ما** | **بعض الشيء** | **بعض الشيء** | **إلى حد ما** | **كثيراً** | **إلى حد كبير** | **كلياً** |

< --------------------**غير صحيح**---------------------><-----------------------**صحيح** ------------------------------------------->

| **1** | **2** | **3** | **4** | **5** | **6** | **7** | **8** | **9** |
| --- | --- | --- | --- | --- | --- | --- | --- | --- |

1. **السجائر** **الالكترونية جيدة المذاق.**

| **1** | **2** | **3** | **4** | **5** | **6** | **7** | **8** | **9** |
| --- | --- | --- | --- | --- | --- | --- | --- | --- |

1. **تدخين السجائر الالكترونية يسيطر على شهيتي**

| **1** | **2** | **3** | **4** | **5** | **6** | **7** | **8** | **9** |
| --- | --- | --- | --- | --- | --- | --- | --- | --- |

| **1** | **2** | **3** | **4** | **5** | **6** | **7** | **8** | **9** |
| --- | --- | --- | --- | --- | --- | --- | --- | --- |

1. **السجائر الالكترونية تساعدني في السيطرة على القلق والتوتر**

| **1** | **2** | **3** | **4** | **5** | **6** | **7** | **8** | **9** |
| --- | --- | --- | --- | --- | --- | --- | --- | --- |

1. **أستمتع بمذاق السجائر الالكترونية**
2. **تدخين السجائر الالكترونية يساعدني على التعامل مع الاكتئاب**

| **1** | **2** | **3** | **4** | **5** | **6** | **7** | **8** | **9** |
| --- | --- | --- | --- | --- | --- | --- | --- | --- |

1. **عندما أدخن السيجارة الالكترونية ، اجد مذاقها مستساغا**

| **1** | **2** | **3** | **4** | **5** | **6** | **7** | **8** | **9** |
| --- | --- | --- | --- | --- | --- | --- | --- | --- |

1. **استمتع بنكهات السيجارة الالكترونية**

| **1** | **2** | **3** | **4** | **5** | **6** | **7** | **8** | **9** |
| --- | --- | --- | --- | --- | --- | --- | --- | --- |

1. **مذاق السيجارة الالكترونية يعطي شعورا جيدا في الفم**

| **1** | **2** | **3** | **4** | **5** | **6** | **7** | **8** | **9** |
| --- | --- | --- | --- | --- | --- | --- | --- | --- |

1. **عند تدخين السجائر الالكترونية ، يزداد خطر اصابتي بأمراض القلب والرئة**

| **1** | **2** | **3** | **4** | **5** | **6** | **7** | **8** | **9** |
| --- | --- | --- | --- | --- | --- | --- | --- | --- |

1. **السجائر الالكترونية تساعدني على التعامل مع المواقف العصيبة**

| **1** | **2** | **3** | **4** | **5** | **6** | **7** | **8** | **9** |
| --- | --- | --- | --- | --- | --- | --- | --- | --- |

1. **عندما اشعر بالضيق من احدهم ، تساعدني السجائر الالكترونية على التأقلم**

| **1** | **2** | **3** | **4** | **5** | **6** | **7** | **8** | **9** |
| --- | --- | --- | --- | --- | --- | --- | --- | --- |

1. **السجائر الالكترونية تبعدني عن تناول الزائد عن الحاجة للطعام**

| **1** | **2** | **3** | **4** | **5** | **6** | **7** | **8** | **9** |
| --- | --- | --- | --- | --- | --- | --- | --- | --- |

1. **السجائر الالكترونية تبقي وزني منخفضا**

| **1** | **2** | **3** | **4** | **5** | **6** | **7** | **8** | **9** |
| --- | --- | --- | --- | --- | --- | --- | --- | --- |

1. **تدخين السجائر الالكترونية يمثل خطرا على صحتي**

| **1** | **2** | **3** | **4** | **5** | **6** | **7** | **8** | **9** |
| --- | --- | --- | --- | --- | --- | --- | --- | --- |

1. **تدخين السجائر الالكترونية يهدئ اعصابي عند الشعور بالتوتر**

| **1** | **2** | **3** | **4** | **5** | **6** | **7** | **8** | **9** |
| --- | --- | --- | --- | --- | --- | --- | --- | --- |

1. **عندما اكون غاضبا ، تدخين السيجارة الالكترونية يهدئ من غضبي**

| **1** | **2** | **3** | **4** | **5** | **6** | **7** | **8** | **9** |
| --- | --- | --- | --- | --- | --- | --- | --- | --- |

1. **تدخين السجائر الالكترونية يجعلني اعيش لسنين أقل**
